# Safe(r) Landing by Older People: A Matter of Complexity

**DOI:** 10.1093/gerona/glae180

**Published:** 2024-07-22

**Authors:** Rich S W Masters, Liis Uiga

**Affiliations:** Te Huataki Waiora School of Health, The University of Waikato, Hamilton, New Zealand; Department of Sport and Exercise Sciences, Manchester Metropolitan University, Manchester, UK

**Keywords:** Degeneracy, Injury reduction, Motor analogies, Unexpected falls

## Abstract

Maintaining balance is a complex motor problem that requires coordinated contributions from multiple biological systems. Aging inevitably lessens the fidelity of biological systems, which can result in an increased risk of falling and associated injuries. It is advantageous to land safely, but falls manifest in diverse ways, so different motor solutions are required to land safely. However, without considerable practice, it is difficult to recall the appropriate motor solution for a fall and then apply it effectively in the brief duration before hitting the ground. A complex systems perspective provides a lens through which to view the problem of safe(r) landing. It may be possible to use motor analogies to promote degeneracy within the perceptual motor system so that, regardless of the direction in which an older person falls, their body self-organizes to land with less likelihood of injury.

For most of us, arriving safely on the ground when we fall is an awkward motor problem, for which an inadequate solution can result in injury or even death. The problem is aggravated by aging, which is associated with progressive multisystem deterioration (ie, degeneration) that affects physical, cognitive, and sensory performance (eg, reduced strength, delayed responses, impaired balance) (1,2). Consequently, countless older adults throughout the world are injured when they fall, taxing healthcare systems with an economic burden that will continue to inflate as the number of older people in the world increases.

The common approach to the problem of falling is to try to lessen fall rates. However, effective fall prevention interventions (eg, exercise/physical therapy programs, Tai Chi) (3,4) tend to be time-consuming, intensive, and challenging to sustain and resource (3,5). Moreover, although evidence suggests that interventions can substantially reduce injury-related consequences of falling (6,7), a recent review and meta-analysis suggested that their efficacy is limited (8). A far less common approach to the problem is to teach people how to land with less likelihood of injury. With much practice, people can master the art of falling. For instance, ukemi (ü-‘ke-mē) is a technique that martial artists practice to limit damage that is caused to them when thrown to the ground by an opponent. Evidence suggests that older people can learn to land more safely by practicing ukemi (9). However, repeatedly landing on the ground to master safe landing is not always feasible for older people, who may neither have the physical resilience nor enough remaining life expectancy to achieve mastery. Recent world guidelines for fall prevention and management do not even list safe landing as a component intervention (3).

How then can we resolve this awkward motor problem so that when older people fall unexpectedly, they are less likely to suffer serious injury? Based on a complex systems perspective, we hypothesize that it may be possible to facilitate the emergence of safe(r) landing behaviors by promoting degeneracy within the motor system.

Degeneracy is considered to be a fundamental property of complex biological systems and refers to the capacity of structurally different constituents of a system to perform the same function or yield the same outcome (10,11). Francis Crick’s Wobble Hypothesis (12), for example, identified that most amino acids can be specified by different codons (trinucleotide sequences of DNA or RNA), and, more relevant to falling, Nicolai Bernstein’s degrees of freedom problem (13) proposed that the “abundance” of ways in which the human body can be configured to move allows the motor system to achieve “repetition without repetition” (14,15). Dancers, for instance, are experts at configuring their bodies to choreograph graceful arrival on the ground when they fall:

I tripped on a crack in the pavement. When I landed, I curved, softened, rolled onto my back, but the contact was mostly down my side … so, you know, I ended up with some scrapes on my elbow and the side of my knee, but nothing twisted, nothing broken. I stood up, went into the restaurant, had a lovely dinner, and walked home hand in hand with my husband.

(Jane—professional dancer)

We propose to achieve this by using motor analogies. In motor learning, analogies have been used to teach beginners the best way to move without overloading them with explicit rules or instructions about movement technique. Analogies leverage familiarity with a concept in one domain to facilitate understanding of a concept in another domain. They allow individuals to effortlessly grasp principles or concepts without being conscious of the rules and knowledge upon which the principles or concepts are based (16). Most drivers, for example, immediately know what it means to “merge like a zip” when they enter the motorway, and if a basketball player has ever taken a cookie from a jar on a high shelf, he or she automatically knows how to execute a free-throw. Evidence not only from sport, but also from surgery, speech therapy, and movement disorders, suggests that motor analogies promote more efficient movement patterns (17–21) and can speed up the learning process (22). By minimizing the amount of information that needs to be explicitly processed during movements, motor analogies may also be well suited to situations in which rapid responses are required (eg, unexpected falls) or where cognitive capacities are diminished (eg, older people). Limited evidence suggests that motor analogies are effective for older people (23–25). For instance, clinically meaningful improvements in walking speed were reported for older people with stroke or Parkinson’s disease when they used motor analogies (24,25).

A motor analogy that encapsulates the concept of safe landing, rather like a simple rule-of-thumb, may provide a method for reducing the severity of injuries to older adults in the event of a fall. For example, “land like you are carrying a baby” communicates how to achieve a safe landing (eg, hold your hands to your chest, fall on your side or back), without the need to explain rules or instructions about technique. Problematically, however, the analogy only provides a solution for forward falls. For other types of falls (backwards/sidewards etc.), a different motor solution may be necessary (26,27). For instance, evidence suggests that sidewards falls are best dealt with by bending the knees and rolling onto the back, whereas backwards falls are best dealt with by squatting (1). A single motor analogy designed to encapsulate correct landing technique is, therefore, unlikely to capture the different movement solutions that are required to achieve a safe(r) landing for each fall type. Multiple analogies, however, are unlikely to be helpful given that there is little time during a fall to recall which landing technique is the most appropriate for the occasion, and then consciously to deploy that strategy when landing.

Given that there is no one safe-landing solution that is applicable in every fall context, we believe that the answer to the problem lies not in providing older people with specific ways to land safely, but rather in promoting, or augmenting, degeneracy that is inherent within the human movement system. A motor analogy that fosters degeneracy within the movement system, that encourages the central nervous system to spontaneously self-organize or configure the most appropriate movement pattern for the occasion, should therefore have greater utility for safe landing in different contexts than different landing techniques that need to be practiced then recalled and executed in the brief time that it takes to fall and hit the ground.

Previous research has shown that instructions to “run softer” or to “make a quieter sound when you land” reduce ground reaction forces when running or jumping (28,29). Studies also indicate that it is possible to reduce fall-related impact forces with “simple” verbal instructions that do not specifically address correct landing technique per se, such as “land with your body relaxed” or “catch the ground” (30). Consequently, we hypothesize that motor analogies that invoke landings that are soft, slow, and silent, potentially will have the power to promote safer landing via any number of the infinite motor solutions that are available to the faller and which are most appropriate for the circumstances of the fall.

Although this hypothesis is speculative, preliminary findings from research conducted in our laboratory suggest that motor analogies potentially are useful for promoting safe(r) landing by young adults (31). Participants with no prior landing-related training were allocated randomly to a motor analogy condition in which they were instructed either to “land like a snowflake” (Experiment 1) or to “land like a feather” (Experiment 2), or a control condition in which they were instructed to “land on the ground” (Experiment 1) or “land safely” (Experiment 2). In Experiment 1, falls were self-initiated forward, backward, or sideward, whereas, in Experiment 2, falls were externally initiated forward, backward, or sideward, with the participant unaware of the direction in which they would fall. Acceleration data from inertial measurement units on different body segments (eg, wrists, head, trunk, etc.) revealed statistically higher free-fall and impact durations and lower maximum acceleration across all sensors for the motor analogy condition compared to the control condition in both experiments. In addition to these parameters, wrist fracture risk ratios (determined in Experiment 2 only) were lower.

These findings are consistent with falls that were soft, slow, and silent, but they do not speak directly to whether motor analogies can yet be considered a multisystem intervention that promotes degeneracy. However, participants instructed to use the motor analogy in our experiments displayed improved landing parameters, irrespective of the different movement configurations elicited by each fall direction (ie, backward/forward/sideward). Further work is needed, however, to quantify the higher-order patterns of behavior that may emerge as a function of degeneracy during landing, perhaps using cluster analysis (32). We might expect to observe safe(r) landing accompanied by more movement clusters in the motor analogy condition compared to the control condition, demonstrating assorted solutions for the same goal, safe(r) landing.

Nevertheless, for now, we believe that it is possible to develop an analogy that is appropriate for many, if not all, fall types because it promotes degeneracy that is inherent in the perceptual motor system. To advance this line of research, a platform of basic science evidence will need to be built using experimental laboratory research to establish the potential benefits of safe-landing analogies for older people—see registered trial, for instance (33)—and then randomized controlled trials will need to be conducted to test the efficacy of the analogies in real-life falling events. Along the way, we will need to examine the extent to which safe-landing analogies generalize across cultures and across different states of aging (eg, frailty, physical health, cognitive functioning, etc.). We will also need to establish the most appropriate ways to embed safe-landing analogies in memory so that they instantaneously are recalled and utilized during a fall, perhaps by practice or visualization or by rooting the analogy when people are very young.

Eventually, safe-landing analogies might be used as a rule-of-thumb that clinicians recommend to older clients or that health authorities use to complement fall prevention programs, casting a wider public health net over people who might fall unexpectedly. Even a marginal reduction in the severity of injuries caused by this complex motor problem has potential for considerable impact on the global health burden in the years ahead.
